# Health-related patterns and chronic kidney disease in the Brazilian population: National Health Survey, 2019

**DOI:** 10.3389/fpubh.2023.1090196

**Published:** 2023-04-06

**Authors:** Letícia Cristina Machado de Sousa, Nathalia Rabello Silva, Catarina Machado Azeredo, Ana Elisa Madalena Rinaldi, Luciana Saraiva da Silva

**Affiliations:** School of Medicine, Federal University of Uberlândia, Uberlândia, Minas Gerais, Brazil

**Keywords:** chronic kidney disease, health behavior, risk factors, factor analysis, public health

## Abstract

**Objective:**

The aim of this study was to identify patterns related to health and their association with chronic kidney disease (CKD) in the Brazilian population.

**Methods:**

We used data from the National Health Survey (PNS), 2019. Participants were interviewed and answered questions related to socioeconomic and demographic information (gender, age, education, race/color), health conditions (presence of hypertension, diabetes mellitus, hyperlipidemia, cardiovascular disease, overweight and CKD) and lifestyle (smoking, alcohol consumption, physical activity and food consumption). To identify patterns, we used exploratory factor analysis. We performed logistic regression models to describe the association of CKD with each pattern in crude models and adjusted for gender, age group, education level and race/color.

**Results:**

A total of 90,846 individuals were evaluated. The prevalence of CKD was 1.49% (95% CI: 1.3–1.6). Three health-related patterns – metabolic factors, behavioral risk factors and behavioral protective factors – were identified by factor analysis. Metabolic factors were determined by the presence of hypertension, diabetes mellitus, hyperlipidemia and cardiovascular diseases. Behavioral risk factors were determined by smoking, alcohol consumption, regular consumption of soft drinks, sweets and artificial juices, and high salt consumption. The protective behavioral factors were established by the practice of physical activity and regular consumption of vegetables and fruits. Participants of the highest tertile for metabolic factors were more likely to have CKD in the adjusted model (OR = 3.61, 95% CI: 2.69–4.85), when compared to those of the lower tertile.

**Conclusion:**

The pattern referring to metabolic factors was associated with a higher chance of presenting CKD.

## 1. Introduction

Chronic kidney disease (CKD) is a significant global health problem. According to the Global Burden of Disease Study, in 2017, 697.5 million cases of all-stage CKD were recorded, for a global prevalence of 9.1% (8.5–9.8) (1). In a systematic review and meta-analysis of observational studies that estimated the prevalence of CKD in general populations, the global prevalence was even higher, 11–13% (2). In 2017, the CKD ranked 12th in the ranking of the cause of mortality with 1.2 million deaths (1) and is expected to jump to 5th place in the ranking of the main causes of early death (3). In Brazil, it is estimated that 3–6 million people have CKD and this disease has as aggravating the fact that it is unknown by many people affected (4, 5).

The rate of CKD may increase in future, not only due to the growth and aging of the population, but mainly due to the increasing prevalence of arterial hypertension (AH), diabetes mellitus (DM), obesity and dyslipidemia (4, 6, 7), which are traditional risk factors for CKD (2, 8–10). Other lifestyle factors, such as smoking, sedentary lifestyle, alcohol abuse, excessive salt consumption and inadequate eating habits have been recognized as important predictors of CKD (9).

In addition to being prevalent, health risk factors are highly interrelated (11). Considering that the simultaneous occurrence of risk factors increases the chance of developing negative health conditions, studies have been concerned with determining how much factors and behaviors are aggregated in individuals. Evidence suggests that the concurrency of different risk behaviors may present a synergistic effect, thus resulting in a multiplicative deleterious effect, rather than an additive effect of each behavior (11, 12).

In this sense, some studies investigated multimorbidity patterns including CKD (13–15), other studies have identified dietary patterns related to CKD (16–19), but none of them included behavioral factors such as smoking, alcohol consumption and physical inactivity, which are also known to be more sensitive to interventions than clinical outcomes. In Brazil, some studies have researched the concurrency of risk behaviors for chronic non-communicable diseases (NCDs) in adults (20–22), however, only three studies identified patterns of risk behavior in a Brazilian national sample (12, 23, 24) and none of them associated these standards with CKD.

Besides, most of these studies performed co-occurrence analyses focusing on competing but independent behaviors. Analyses investigating underlying associations between competing behaviors, with clustering identified by divergences in observed and expected prevalence of combinations or by identifying latent or unobservable patterns (25), may be more relevant from the point of view of monitoring and planning of more effective multifactorial interventions (11), which should take into account substitute and complementary relationships between grouped health behaviors.

Thus, the aim of the study was to identify patterns related to health and their association with CKD in the Brazilian population.

## 2. Methods

### 2.1. Study population and sampling

The present study used data from the National Health Survey (PNS), a Brazilian household survey conducted in 2019 by the Brazilian Institute of Geography and Statistics (IBGE) in partnership with the Health Surveillance Secretariat of the Ministry of Health and the Oswaldo Cruz Foundation.

The sampling plan was defined by conglomerate in three stages of selection. In the first stage, the primary sampling units were stratified, consisting of sectors or groups of census tracts. The second stage consisted of households, and residents 15 years of age or older corresponded to the units of the third stage. The selection of a subsample of the primary sampling units was made by simple random sampling (26).

The estimated sample size was based on 108,525 households, and 94,114 individuals aged 15 years or older were interviewed. More details about the research are available in the publication of STOPA et al. (26)

### 2.2. Data collection and studied variables

Participants were interviewed and answered questions related to socioeconomic, demographic information, health conditions and lifestyle. The socioeconomic and demographic variables evaluated were: gender (male and female); age categorized into groups (15–29; 30–44; 45–59; 60 or more); education (without incomplete education/elementary school; complete elementary school/incomplete high school; complete high school/incomplete higher education; complete higher education) and race/color [white; black; brown and others (yellow and indigenous)].

Information on AH, DM, CKD, hypercholesterolemia and cardiovascular disease was based on self-reported previous medical diagnosis. In the identification of overweight, body mass index (BMI) was calculated according to self-reported weight and height. The cutoff point for overweight was BMI ≥ 25.0 kg/m^2^ for adults (27) and BMI ≥ 27.0 kg/m^2^ for the elderly (28).

Among the risk and protection factors related to lifestyle, we analyzed smoking (non-smoker, former smoker and smoker), alcohol consumption (heavy episodic drinking), physical activity and food consumption. Alcohol consumption, considering heavy episodic drinking, was defined as the intake of 60 g or more of alcohol (five or more doses of alcohol) on at least one occasion in the last 30 days (29). Two questions were used: “How often do you usually consume an alcoholic beverage? (I never drink, less than once a month and once or more a month)” and “In the last 30 days, did you consume five or more doses of alcoholic beverages on a single occasion? (yes and no).” A dose of drink was defined as the equivalent of a dose of cachaça, a glass of wine, a can of beer, a dose of whiskey or any other distilled alcoholic beverage. Heavy episodic drinking was considered when individuals answered “once or more a month” in the first question and “yes” in the last question. For physical activity, individuals who practiced at least 150 min of physical activity per week were considered active, considering four domains: leisure, work, commuting and domestic activities (30). Regarding food consumption, the frequency of weekly consumption of soft drinks, artificial juices, sweets (such as biscuit/stuffed cookie, chocolate, gelatin, candies and others), fruits or vegetables (such as lettuce, tomato, cabbage, carrot, chayote, eggplant, zucchini, excluding potatoes, cassava or yams) and beans were analyzed. Food consumption was considered regular when it occurred five or more times a week (6).

The perception of salt consumption was obtained considering the following question: “Considering freshly prepared food and processed foods, you think your salt intake is”: very high, high, adequate, low and very low. Salt intake was categorized as adequate (adequate, low and very low) and high (high and very high).

### 2.3. Statistical analysis

For descriptive analysis, the following variables were considered, according to the presence of CKD: gender, age group, education level, race/color, AH, DM, hypercholesterolemia, cardiovascular disease, overweight, physical activity, smoking, alcohol consumption, regular consumption of beans, regular consumption of vegetables, regular consumption of fruits, regular consumption of soft drinks, regular consumption of artificial juices, regular consumption of sweets and high salt consumption. The chi-square test was used to verify whether there is a statistically significant difference between individuals with and without CKD.

To identify patterns, we used exploratory factor analysis to reduce the initial number of variables in a smaller set of factors that represent, in a synthetic way, the information contained in the larger set of variables (31).

The adequacy of the data for factor analysis was initially evaluated with the Kaiser-Meyer-Olkin (KMO) sample adequacy measure. KMO assumes values between 0 and 1, and the lower values indicate that the variables have very little in common. A value of KMO = 0.62 was obtained, which means good adequacy (32).

The principal component analysis was used to extract the factors and the oblique promax rotation was performed to facilitate the interpretation of the factors. The determination of the number of factors to be retained considered the following criteria: the evaluation of the scree plot, the factor structure with loads of items above 0.30 (33), the lowest number of cross-loads of possible items, no factor with less than three items and a reasonable interpretation of the emerging factors.

Then, we identified three health-related patterns, named metabolic, risk behavior and protective behavior. Each individual had values assigned to each of the patterns, through regression models, according to their higher or lower adhering to the pattern. The values of each pattern were categorized into tertiles (the lowest category and the highest category represent the lowest and highest adhesion to a specific pattern, respectively). The first tertile of each pattern was used as a reference group.

Subsequently, we performed logistic regression models to describe the association of CKD with each pattern in crude models and adjusted for gender, age group, education level and race/color.

The analyses were performed in Stata software version 14.2, considering significance level of 5% and the effects of complex PNS sampling.

### 2.4. Ethical aspects

The PNS project was approved by the National Research Ethics Commission (CONEP) of the National Health Council (CNS) (opinion n. 3529376). The invited individuals who agreed to participate in the research signed the Free and Informed Consent Form.

## 3. Results

A total of 90,846 individuals were evaluated. The prevalence of CKD was 1.49% (CI 95%: 1.3–1.6). Regarding the characterization of the population studied, it is noteworthy that the majority of the study participants were women, aged between 30 and 44 years, brown and had completed high school education. A higher prevalence of CKD was found in older individuals (*p* < 0.001), with lower education level (*p* < 0.001) and white (*p* = 0.015; Table 1).

**Table 1:** Sociodemographic characteristics and prevalence of metabolic and behavioral factors, according to the presence of CKD. PNS, 2019.

	Variables	Total	CKD		Value of *p*
			No	Yes	
		% (CI 95%)	% (CI 95%)	% (CI 95%)	
Gender	Male	47.1 (46.8–47.4)	47.1 (46.8–47.5)	45.4 (42.7–48.1)	0.2
	Female	52.9 (52.6–53.2)	52.9 (52.5–53.2)	54.6 (51.9–57.3)	
Age group	15–29 years	19.5 (19.2–19.7)	19.6 (19.4–19.9)	7.4 (6.1–9.0)	**<0.001**
	30–44 years	29.4 (29.1–29.7)	29.6 (29.3–29.9)	19.3 (17.2–21.5)	
	45–59 years	26.0 (25.7–26.3)	25.9 (25.7–26.2)	32.2 (29.7–34.8)	
	60 years or more	25.0 (24.7–25.3)	24.8 (24.5–25.1)	41.1 (38.4–43.8)	
Education level	Uneducated/incomplete elementary school	33.1. (32.5–33.8)	34.1 (33.5–34.8)	46.7 (42.2–51.3)	**<0.001**
	Complete elementary school/incomplete high school	17.1 (16.6–17.6)	17.4 (17.0–17.9)	17.8 (14.3–22.0)	
	Complete high school/incomplete higher education	34.1 (33.5–34.6)	33.4 (32.8–33.9)	25.1 (21.4–29.2)	
	Complete higher education	15.7 (15.1–16.3)	15.0 (14.4–15.6)	10.4 (7.9–13.5)	
Race/color	White	43.6 (42.8–44.3)	42.9 (42.1–43.6)	48.2 (43.6–52.8)	**0.015**
	Black	11.2 (10.8–11.6)	11.4 (11.0–11.8)	10.0 (7.4–13.4)	
	Brown	43.7 (43.0–44.3)	44.2 (43.6–44.9)	40.4 (36.1–44.8)	
	Others (indigene or yellow)	1.5 (1.3–1.7)	1.5 (1.3–1.7)	1.4 (0.8–2.3)	
Arterial hypertension		24.8 (24.3–25.2)	23.9 (23.4–24.3)	53.7 (49.1–58.3)	**<0.001**
Diabetes mellitus		8.0 (7.7–8.3)	7.7 (7.5–8.0)	21.3 (17.8–25.2)	**<0.001**
Hypercholesterolemia		14.8 (14.4–15.2)	14.5 (14.1–14.9)	33.4 (29.0–38.1)	**<0.001**
Cardiovascular disease		5.2 (5.0–5.5)	4.8 (4.6–5.1)	20.5 (16.8–24.7)	**<0.001**
Overweight		61.6 (61.0–62.2)	61.6 (61.0–62.2)	64.7 (60.2–69.0)	0.174
Physically active		46.3 (45.6–47.0)	46.2 (45.6–46.8)	34.6 (30.4–39.2)	**<0.001**
Smoking		11.4 (11.0–11.8)	12.1 (11.8–12.5)	12.1 (9.3–15.6)	0.976
Alcohol consumption		14.2 (13.8–14.6)	14.6 (14.1–15.0)	9.4 (6.8–12.7)	**0.004**
Regular consumption of beans		68.0 (67.4–68.7)	68.2 (67.6–68.9)	63.5 (58.9–68.0)	**0.039**
Regular consumption of vegetables		55.1 (54.4–55.7)	54.0 (53.4–54.7)	59.2 (54.7–63.6)	**0.026**
Regular consumption of fruits		45.4 (44.7–46.0)	44.2 (43.6–44.8)	48.1 (43.6–52.6)	0.087
Regular consumption of soda		9.2 (8.9–9.6)	9.6 (9.2–10.0)	7.8 (5.4–11.2)	0.265
Regular consumption of artificial juices		13.3 (12.8–13.7)	13.8 (13.4–14.3)	11.0 (8.2–14.5)	0.11
Regular consumption of sweets		15.4 (14.9–15.9)	15.7 (15.2–16.2)	13.1 (10.1–16.8)	0.164
High salt consumption		12.5 (12.0–13.0)	12.9 (12.4–13.4)	11.2 (8.6–14.4)	0.279

Bold data reflect statistical significance (*p* < 0.05).

Among individuals with CKD, it was possible to observe a higher prevalence of AH (53.7%), DM (21.3%), hypercholesterolemia (33.4%), cardiovascular disease (20.5%) and lower physical activity (34.6%; Table 1).

Alcohol consumption and regular consumption of beans were more prevalent in the group of participants without CKD, while regular consumption of vegetables was higher in the group of people with CKD (Table 1).

Three health-related patterns – metabolic factors, behavioral risk factors and behavioral protective factors – were identified by factor analysis. These patterns explained 11.25, 9.34 and 9.33% of variance, respectively (Table 2). Together, they explained ~30% of the variance.

**Table 2:** Factor structure of metabolic, behavior risk and behavior protective in the Brazilian population. PNS, 2019.

Factors	Metabolic	Behavior risk	Behavior protective
Arterial hypertension	**0.7166**	−0.0271	0.0364
Diabetes mellitus	**0.6091**	−0.0362	0.0254
Hypercholesterolemia	**0.6186**	0.0565	0.1010
Cardiovascular disease	**0.4982**	0.0746	0.0688
Overweight	0.2473	0.0132	−0.1935
Smoking	0.0136	**0.3949**	−0.1658
Alcohol consumption	−0.1092	**0.4516**	−0.0188
Regular consumption of soda	0.0097	**0.5671**	−0.0515
Regular consumption of sweets	0.0184	**0.4868**	0.1856
Regular consumption of artificial juices	0.0218	**0.4354**	−0.0086
High salt consumption	0.0431	**0.4762**	−0.0731
Regular consumption of beans	0.0230	0.0462	0.1012
Regular consumption of vegetables	0.0605	0.0385	**0.7417**
Regular consumption of fruits	0.1034	−0.0956	**0.7392**
Physically active	−0.2609	0.1064	**0.4056**
Variance explained (%)	11.25	9.34	9.33
Cumulative variance (%)	11.25	20.59	29.92

Bold data reflect that the variable has loaded in the pattern.

Regarding the characterization of these patterns, metabolic factors were determined by the presence of AH, DM, hyperlipidemia and cardiovascular diseases. Behavioral risk factors were determined by smoking, alcohol consumption, regular consumption of soda, regular consumption of sweets, regular consumption of artificial juices and high salt consumption. The behavioral factors of protection were established by the practice of physical activity, regular consumption of vegetables and regular consumption of fruits. The highest factorial loads were found in the metabolic pattern for AH, DM and hypercholesterolemia and in the behavior protective pattern for regular consumption of vegetables and fruits (Table 2). Higher factor loadings indicate greater adherence to the respective pattern.

The association of the patterns identified with CKD, stratified by tertiles, were presented in Table 3. Participants of the highest tertile for metabolic factors were more likely to have CKD in the crude model (OR = 4.39, 95% CI 3.33–5.79) and adjusted (OR = 3.61, 95% CI 2.69–4.85), when compared to those of the lower tertile. No significant association was observed between behavioral risk and protection factors and the presence of CKD.

**Table 3:** Association of the three health-related patterns with CKD, by tertile. PNS, 2019.

	Crude model			Adjusted model^a^		
	OR (CI 95%)			OR (CI 95%)		
	1st tertile	2nd tertile	3rd tertile	1st tertile	2nd tertile	3rd tertile
Metabolic factors	1	1.18	4.39	1	1.11	3.61
		(0.84–1.66)	(3.33–5.79)		(0.79–1.56)	(2.69–4.85)
Behavioral risk factors	1	0.98	0.8	1	1.03	0.98
		(0.78–1.22)	(0.63–1.02)		(0.82–1.29)	(0.77–1.24)
Behavioral protective factors	1	1.01	1	1	0.96	0.89
		(0.80–1.28)	(0.79–1.27)		(0.76–1.22)	(0.70–1.14)

a Adjusted for gender, age group, education level and race/color.

## 4. Discussion

The findings of the present study showed a prevalence of CKD of 1.49%. In the factor analysis, three health-related patterns were identified, labeled as: metabolic factors, behavioral risk factors and behavioral protective factors. Only metabolic factors were associated with the chance of presenting CKD.

The prevalence of CKD in the Brazilian population is still uncertain (5). According to PNS data, the prevalence of self-reported CKD was 1.4% (34), as described in the present study. In a subsample of the PNS, in which laboratory tests were performed, the prevalence of CKD was 6.48% (35). The prevalence of CKD among participants of the Longitudinal Study of Adult Health (ELSA), in six research institutions in Brazilian capitals, was 8.9% (36). The difference in self-reported prevalence and that assessed by laboratory tests shows the high percentage of unknown cases of the disease. The hidden cases of the disease can be explained by the lack of screening for the disease and by the insidious and asymptomatic loss of renal function, being a major public health problem.

About the health-related patterns, Corsonello et al. (13) reported that CKD is associated with multimorbidity and was rarely observed without any concomitant disease, and AH and DM were among the co-ocurrent pairs of greater significance involving CKD. In addition, it is known that these factors are the main causes of CKD worldwide (37). In agreement, this study showed the importance of metabolic factors, which include AH and DM, in the development of CKD.

AH may be the cause or consequence of CKD. In hypertensive patients, chronically increased systemic arterial pressures cause remodeling of the aferent arteriola and reduce its capacity for contraction and dilation. Over time, increased blood pressure and pressure transmitted to the kidney lead to nephrosclerosis and progressive loss of renal function (38).

With regard to DM, chronic hyperglycemia leads to metabolic dysregulation due to increased glycolysis, which regulates several distinct pathways and leads to glomerular hyperfiltration and proteinuria. In addition, hyperglycemia causes hemodynamic changes in the kidney, oxidative stress, inflammation, hypoxia and deregulation of the Renin-Angiotensin-Aldosterone system (RAAS), which causes adverse changes in the kidney vessels, such as thickening of the glomerular basement membrane (39–42).

In the study by Liu et al. (43), the relationship between cardiovascular diseases and CKD was demonstrated, evidencing a deep association between them, and the disease of one organ causes dysfunction in the other. Thus, it is assumed that two main mechanisms can explain this association. The kidney can release hormones (44, 45), enzymes and cytokines (44, 46) in response to kidney injury, which leads to changes in blood vessels. In addition, CKD mediators and hemodynamic changes contribute to heart damage (44, 47).

Likewise, in the study by Chang et al. (48) it has been reported that older adults with hyperlipidemia and cardiovascular diseases were at higher risk of developing CKD. This association is due to the fact that patients with CKD tend to present physiological and biochemical alterations that lead to imbalance in lipid profile. In addition, triglyceride levels are increased by 30–50%, which is related to the reduction of hepatic lipase and lipoprotein lipase activity. There is a decrease in HDL-cholesterol, increased lipoprotein A and the accumulation of LDL-cholesterol. Thus, there is a prevalence of oxidized LDL molecules, which are captured by immune system cells, with consequent contribution in the formation of atherosclerotic plaque. Another mechanism related to dyslipidemia is its ability to damage mesangial and endothelial cells, which facilitates the progression of kidney injury. The mechanisms involving dyslipidemia and CKD are not yet fully understood, but it is possible to highlight some more relevant factors such as insulin resistance and increased oxidative stress (49, 50).

The findings of this study allowed the identification of a higher chance of having CKD for those individuals in the highest tertile of the pattern called metabolic factors. Therefore, the importance of evaluating these factors simultaneously is highlighted, because the individual hardly has only one isolated factor and this demonstrates the role of multimorbidity in this disease (51, 52).

In relation to the other patterns analyzed, it is noteworthy that this study did not observe a significant association of behavioral risk factors and behavioral factors of protection with CKD, similar to the results presented by Foster et al. (53), who found no association between CKD and physical activity, smoking and alcohol consumption. In the Brazilian context, study with a representative sample of the population, in univariate analysis, found an association between CKD and some behavioral factors, such as smoking, excessive alcohol consumption and physical activity, but also found no association between CKD and regular intake of fruits and vegetables, consumption of red meat with fat and excessive consumption of salt. In an adjusted analysis, considering behavioral factors, only smoking remained associated with CKD (35).

However, other studies have shown that behavioral factors may influence the presence and development of CKD (13, 16, 18, 24, 48, 54). Regarding the association of dietary patterns with CKD, studies conducted with the populations of Iran (16), United Kingdom (55) and China (19, 56) found that dietary patterns high in fat and sugar were associated with higher chances of incidence of CKD, while plant-rich dietary patterns were associated with reduced risk of CKD. However, due to the diversity of eating habits in the world, the results found in these studies cannot be extrapolated to other populations (17). In addition, the absence of association of behavioral factors with CKD in the present study may be due to cross-sectional design and reverse causality, as lifestyle risk and protection behaviors may be altered due to the diagnosis and guidance given by health professionals to delay the progression of CKD.

Although patterns related to risk and protective factors have not been associated with CKD, it can be inferred as a practical application the encouragement of protective behaviors and the reduction of risk behaviors. Thus, investing in NCD prevention actions can be a good strategy to prevent the development and progression of CKD. In this sense, another practical application is the strengthening and implementation public policies aimed at CKD with the objective of encouraging the promotion of healthy habits, which makes it possible to prevent the occurrence of NCDs, such as AH and DM, and, consequently, CKD.

In Brazil, the implementation of public policies for the prevention and management of kidney diseases is recent and incipient (57). In 2004, the *National Policy for Attention to Patients with Kidney Disease* was instituted by Ordinance n° 1.168/2004. In 2006, the Ministry of Health published guidelines for the *Clinical Prevention of Cardiovascular, Cerebrovascular and Chronic Kidney Disease*, which recommended early screening in primary care in risk groups, such as AH, DM and family history of CKD. In 2014, the Ministry of Health published Ordinance n° 389/2014, which defined the criteria for the organization of the line of care for people with CKD and published the *Clinical Guidelines for the Care of Patients with Chronic Kidney Disease in the Sistema Único de Saúde (SUS)*. In addition, reinforcing that the main action in the prevention of CKD cases is the reduction and treatment of the main risk factors for the development of kidney disease, in 2011, the Federal Government prepared the *Strategic Action Plan to Combat Chronic Noncommunicable Diseases in Brazil 2011–2022*, which was recently updated, considering the period 2021–2030 (57).

Despite all the efforts made toward the reduction of chronic conditions, it is observed that challenges still need to be overcome to ensure improved care for people with CKD. A recent study shows flaws in the screening of people at risk for CKD in primary health care in Brazil (58) and worldwide (59). It also highlights the need for actions to improve the control of the most prevalent conditions in the adult population, that is, AH, DM and dyslipidemia (58, 59). Therefore, it is necessary to implement CKD control and prevention strategies, which consist of the quality and effectiveness of existing programs in primary care, as well as the degree of motivation, training and continuing education of health professionals (57).

This study has strengths and limitations. Regarding the strengths, the sample size of the study stands out, being representative of the Brazilian population. In addition, different factors were analyzed: metabolic, risk and protective behavior. Moreover, this study used factor analysis, a statistical analysis technique that allows grouping the variables, according to the correlations between them, that is, with this analysis the pattern of the variables and the adhering to this pattern by the individuals is better represented, surpassing the analysis of the occurrence of isolated factors or the co-occurrence of factors.

As for limitations, it is found that the design of the cross-sectional study does not allow establishing a causal relationship. Consequently, the possibility of reverse causality also exists. Future studies using prospective cohorts are needed to explain and confirm a causal relationship between health-related patterns and CKD. In addition, there may have been interviewer bias and participants’ memory bias, since the data were self-reported. Also noteworthy is the possibility of underdiagnosis, especially of metabolic factors, leading to underestimated prevalence. Regarding the excessive salt consumption variable, the results of the present study cannot be seen as an approximation of the real salt consumption by the Brazilian adult population, since the agreement between the perceived and actual level of salt consumption is distorted (Brazilian consume, on average, almost twice the World Health Organization recommendations, yet a small fraction acknowledge their excessive intake) (60). Finally, it was not possible to stratify the severity of the disease.

Accordingly, our study made it possible to identify that the pattern referring to metabolic factors was associated with a higher chance of presenting CKD, while patterns related to behavioral risk factors and behavioral protective factors were not significantly associated. This suggests that underlying diseases, such as AH and DM, may be more strongly linked to the chance of CKD than behavioral factors such as diet and physical activity.

## Data availability statement

Publicly available datasets were analyzed in this study. This data can be found here: https://www.ibge.gov.br/estatisticas/sociais/saude/9160-pesquisa-nacional-de-saude.html?=&t=downloads.

## Ethics statement

The 2019 National Health Survey project was forwarded to the National Research Ethics Committee (CONEP)/National Health Council (CNS) and approved under Opinion No. 3,529,376, issued on 23 August 2019. The participants provided their written informed consent to participate in this study.

## Author contributions

LCMS and NRS contributed to the formal analysis of the data. CMA, AEMR, and LSS contributed to conceptualization and visualization, formal analysis of the data. All authors contributed to the article and approved the submitted version.

## Conflict of interest

The authors declare that the research was conducted in the absence of any commercial or financial relationships that could be construed as a potential conflict of interest.

## Publisher’s note

All claims expressed in this article are solely those of the authors and do not necessarily represent those of their affiliated organizations, or those of the publisher, the editors and the reviewers. Any product that may be evaluated in this article, or claim that may be made by its manufacturer, is not guaranteed or endorsed by the publisher.
